# Characteristics and Reversibility of Dementia in Normal Pressure Hydrocephalus

**DOI:** 10.1155/2007/456281

**Published:** 2007-08-22

**Authors:** Priyanka Chaudhry, Siddharth Kharkar, Jennifer Heidler-Gary, Argye E. Hillis, Melissa Newhart, Jonathan T. Kleinman, Cameron Davis, Daniele Rigamonti, Paul Wang, David N. Irani, Michael A. Williams

**Affiliations:** Johns Hopkins UniversitySchool of MedicineBaltimoreMDUSA

## Abstract

Studies of the cognitive outcome after shunt insertion for treatment of Normal Pressure Hydrocephalus have reported widely mixed results. We prospectively studied performance of 60 patients with Normal Pressure Hydrocephalus on a comprehensive battery of neuropsychological tests before and after shunt surgery to determine which cognitive functions improve with shunt insertion. We also administered a subset of cognitive tests before and after temporary controlled drainage of cerebrospinal fluid to determine if change on this brief subset of tests after drainage could predict which patients would show cognitive improvement three to six months after shunt insertion. There was a significant improvement in learning, retention, and delayed recall of verbal memory three to six months after surgery (using paired t-tests). The majority (74%) of patients showed significant improvement (by at least one standard deviation) on at least one of the memory tests. Absence of improvement on verbal memory after temporary drainage of cerebrospinal fluid had a high negative predictive value for improvement on memory tests at 3–6 months after surgery (96%; *p* = 0.0005). Also, the magnitude of improvement from Baseline to Post-Drainage on few specific tests of learning and recall significantly predicted the magnitude of improvement after shunt surgery on the same tests (*r*^2^ = 0.32–0.58; *p* = 0.04–0.001). Results indicate that testing before and after temporary drainage may be useful in predicting which patients are less likely to improve in memory with shunting.

## 1. Introduction

Normal pressure hydrocephalus (NPH) is a treatable neurological disorder characterized by abnormal gait, urinary incontinence, and dementia/cognitive deficits that generally occurs in the sixth and seventh decades of life [1,11,25]. Imaging shows enlargement of the ventricles along with compression or thinning of the corpus callosum. NPH is thought to account for as much as 5% of all dementias [33]. The incidence has been reported as 1.8 out of 100,000 people, although this number may be an underestimate, as many cases may not be recognized [33]. Symptom onset is generally gradual.

The dementia of NPH is characterized by subcortical cognitive deficits, involving psychomotor slowing, impaired recall, and impaired executive functions. These deficits are often mistaken for the consequence of old age. There is a gradual decline in active retrieval from memory (immediate and delayed recall) with relatively preserved memory storage (recognition). Furthermore, there is decline in executive functions and complex information processing (increased reaction time, impaired ability to manipulate acquired knowledge, and decreased cognitive flexibility) [33]. Sometimes there are also deficits in visuospatial perception and visual construction skills. A recent study compared 21 NPH patients with 42 Alzheimer's disease (AD) patients matched in age, sex, and Mini-Mental State Examination score, on standardized neuropsychological tests. The NPH group scored significantly higher on the orientation subtest of the Alzheimer's Disease Assessment Scale and on the general memory and delayed recall subtests of the Wechsler Memory Scale-

ISSN 0953-4180/07/$17.00 © 2007 – IOS Press and the authors. All rights reserved

Revised (WMS-R), but significantly lower on the attention/concentration subtest of the WMS-R, and on the digit span, arithmetic, block design and digit symbol subtests of the Wechsler Adult Intelligence Scale-Revised [20].

There is broad consensus that at least the physical symptoms of NPH are relieved by cerebrospinal fluid (CSF) removal by large volume lumbar puncture or more permanently with insertion of a ventriculoperitoneal (VP) or ventriculoatrial shunt [3,17]. In many cases, the most dramatic improvement is seen in gait. Studies report variable results regarding the reversibility of the cognitive deficits after shunt surgery [2,4,6, 15,16,23,24,26,31,32]. A recent study compared subjective outcomes of 50 patients at three months versus three years after shunt surgery. Outcomes were classified as favorable (excellent/good or partial recovery) versus poor recovery. Favorable recovery of gait was present for 91% at three months and declined to 75% at three years. In contrast, memory improvement had 80% favorable recovery at both time points, and incontinence increased from 70% favorable at 3 months to 82.5% at three years after shunt surgery [2].

Another study compared change in cognitive function and ambulation in nine patients with idiopathic NPH who underwent shunt insertion and 10 who did not. Shunted patients were studied pre-operatively and at a mean of 37 months after shunt insertion; controls were studied at two comparable time points. Improved gait or psychometric function were found in the majority of the shunted patients but none of the control patients ($p < 0.02$) [26]. In a study 56 NPH patients with poor prognostic factors (idiopathic type, cortical atrophy, longstanding symptoms, presence of dementia, and old age), gait improved in 100% of patients, sphincter control improved in 90%, and dementia (assessed with Mini Mental State Examination and other tests of verbal and visual memory) improved in 33%. Significant improvement was also found in most daily life activity scales after shunt surgery [23].

Previous studies of the cognitive outcome after shunt surgery have reported widely mixed results. A 1978 reported improvement in 12/23 patients with hydrocephalic dementia [10]. A 1989 study of 30 patients shunted for NPH showed a positive effect on cognitive functions of distractibility of attention and motor speed, but not on intelligence or memory Sixteen showed significant improvement on neuropsychological tests (mainly those for attention, motor speed and memory), while three patients deteriorated and eleven remained stable. They also reported that response to large volume lumbar puncture had no predictive value for improvement in neuropsychological functions after shunting [32]. A larger study of 74 patients followed prospectively for an average of 2.1 years documented that prior to treatment 96% of patients had cognitive symptoms, 95% had gait disturbance, and 75% had incontinence. Improvement in one or more domains was observed in 78% after shunt surgery, while 22% deteriorated. Improvement in cognitive function was seen in 80% and improvement in gait in 76% [15]. Another study reported that 66.6% of patients improved (24% markedly and 43% moderately) in cognition after shunting [24]. Still another study of 35 patients demonstrated that 37% had improved spatial function, while 29% showed improvement in object memory three months after shunt [16]. Again, response to lumbar puncture did not predict which patients would improve. Thus, some improvement has been reported in 52–80% of patients who undergo shunt insertion for NPH. However, these studies used a limited number of cognitive tests or subjective scales.

Several studies have used a more comprehensive battery of neuropsychological tests to evaluate response. One showed no significant improvement in any of the neuropsychological tests administered after shunt surgery [27]. In a more recent study, there was significant overall neurocognitive improvement in 22/42 patients (52.3%) three months after shunt surgery [31]. 14/27 patients (51.8%) improved significantly in 50% or more of the administered neurocognitive subtests of an extensive battery, and 8/15 (53%) studied only with the Mini Mental State Examination improved significantly on this test.

In all of the studies to date, only a subset of treated patients has shown improvement in cognition after shunt surgery. Factors that may affect cognitive outcome include age, associated medical conditions such as diabetes mellitus and cerebrovascular disease, and gender. Our group studied 36 patients to identify the demographic factors associated with cognitive improvement after shunt insertion [4]. We found that there were better outcomes for younger patients, and this relationship was linear. Also, women had more cognitive improvement than men. Age was found to be a better predictor of improvement on memory tests, while sex was a better predictor of improvement on non–memory tests. Another potential predictor of outcome is response to temporary CSF drainage via spinal catheter. As noted earlier, a few studies have indicated that response to lumbar puncture is not a strong predictor. Therefore, recent studies have evaluated response to 2–4 days of controlled, continuous CSF drainage, which comes closer to simulating the effect of a shunt. Patients who improve in either cognition or gait after CSF drainage are considered to be good candidates for shunt surgery. Therefore we tested the hypothesis that patients who respond well on neurocognitive testing after a controlled CSF drainage trial will respond well after shunt surgery.

We prospectively evaluated changes in performance on a comprehensive battery of tests before and after shunt surgery in a large cohort ($n = 60$). We evaluated whether improvement in any of the subtests administered after 2–3 days of controlled CSF drainage predicts cognitive improvement after shunt surgery. To accomplish this, we administered a cognitive screening battery after the controlled CSF drainage trial that consisted of a subset of the larger battery of tests given before and at three months after surgery. We hypothesized that the magnitude of cognitive improvement from the Baseline to Post-Drainage conditions would predict cognitive improvement three to six months after shunt surgery.

## 2. Methods

### 2.1. Patients

From 2000–2005, a series of 60 patients with NPH underwent neurocognitive testing at baseline and three months after shunt insertion. Patients were evaluated for NPH if they had two or more symptoms of the NPH triad: cognitive impairment, gait disturbance, or urinary incontinence. All patients had enlarged ventricles on computed tomography or magnetic resonance imaging. Exclusion criteria were: a known cause for hydrocephalus, associated medical conditions that would prevent cognitive testing (e.g., blindness, deafness). Thus, all patients met the criteria for possible or probable idiopathic NPH at the time of evaluation [25].

### 2.2. Procedure

Patients with suspected NPH were admitted to the Johns Hopkins Hospital for controlled CSF drainage via spinal catheter to assess symptom response, and to determine if they were likely to benefit from a shunt [31].

Patients and their families answered a comprehensive questionnaire regarding their symptoms at the time of admission, and at each follow-up visit. In particular regard to their perception of cognitive impairment at baseline they were asked the Yes/No question, "In the past month, did you have trouble with your thinking or memory?" In regard to their perception of cognitive change after shunt surgery, they were asked, "Overall, as compared to the time before your shunt surgery, how is your thinking and memory?" Responses for this question were scored on a 5-point Likert scale. For analysis, the "Better" and "Much better" responses were recoded as "Yes" and all other responses were recoded as "No".

Neurocognitive tests were administered at 3 test conditions: (1) hospital admission (Baseline), (2) after the CSF drainage trial (Post-Drainage), and (3) 3–6 months after shunt surgery (Post-Shunt). Some patients did not receive Post-Drainage testing because they were discharged before testing could be done, because the patient declined the testing, or because a complication prevented completion of controlled lumbar drainage. Therefore, 40 of 60 patients had the Post-Drainage testing. Also, some patients were unable to complete all of the tests at one or more of the test conditions; scores on the incomplete tests were treated as missing values.

### 2.3. Tests administered

The cognitive battery administered at Baseline and at three to six months after shunt surgery included: 1) Wechsler Memory Scale logical memory subtest, testing verbal recent memory (immediate and delayed recall of story) [34]; 2) Rey –Osterreich's Complex Figure test, testing recent visual memory (copy, immediate recall, and delayed recall) [21]; 3) Rey Auditory Verbal Learning Test (RAVLT) testing learning abilities [28]; 4) Boston Naming Test, testing picture recognition and word retrieval [9]; 5) timed writing of the alphabet, testing graphomotor skills and psychomotor speed; (6) timed copying of pentagons, testing visuospatial performance and psychomotor speed; 7) Grooved Pegboard Test (Psychological Assessment Resources, Inc., Lutz, Fla), testing fine motor speed; 8) Trail Making Test, Parts A and B, testing visuospatial performance, executive function, and psychomotor speed [22]; 8) Stroop Color Task and Stroop Color-Word test [8], testing ability to inhibit the first response, cognitive flexibility, and intellectual speed; 9) Controlled Oral Word Association Test (COWA), testing word fluency and speed of executive functions [30]; 10) Line tracing, testing psychomotor speed and fine motor control. Alternative forms of Wechsler Memory logical memory test, COWA, and RAVLT were administered at Baseline and

Post-Shunt to minimize practice effects. The Boston Naming Test and RAVLT recognition memory subtest were included to detect the presence of a cortical dementia such as Alzheimer's disease; scores were not expected to be abnormal in "pure NPH" and were not expected to improve with shunt surgery.

The subset of tests done after the controlled CSF drainage trial included the RAVLT, COWA, alphabet writing, line tracing and copying pentagons.

### 2.4. Analysis of results

Variation of patients' scores from normal (in standard deviations, SD) was based on published norms from healthy adults for WMS, Rey Complex Figure test, RAVLT, Trail Making test, and Stroop Color-Word tests. Data analyses included paired t-tests using age-adjusted scores at Baseline and Post-Shunt. Performance was considered definitely abnormal for scores more than 2 SD below the mean; borderline abnormal for scores 1–2 SD below the mean; and normal for scores higher than 1 SD below the mean for the patient's age at the Baseline, Post-Drainage, and Post-Shunt conditions. To determine if the magnitude of cognitive improvement from Baseline to Post-Drainage correlated with the magnitude of improvement from Baseline to Post-Shunt, we calculated Pearson correlations. We also calculated the positive and negative predictive value of a five point improvement in RAVLT learning scores from Baseline to Post-Drainage for predicting improvement in more than half of memory tests at three to six months Post-Shunt surgery. To assess the relationship between cognitive testing results and patient responses regarding their perception of baseline cognitive impairment, or change in cognitive function after shunt surgery, receiver operating curve (ROC) analysis was used.

## 3. Results

The mean age was 70.6 ± 12.1 years and mean duration of symptoms was 3.5 years. There were 24 women and 36 men.

There were significant improvements from Baseline to Post-Shunt on: WMS Delayed Recall ($t = -2.13$; df49; $p = 0.04$), RAVLT learning (RAVLT total score for recall of a word list over five trials; $t = -3.3$; df55; $p = 0.002$), RAVLT retention (recall of the initial RAVLT list after learning an "interference" list; $t = -2.7$; df55; $p = 0.009$), RAVLT delayed recall ($t = -4.2$, df55; $p = 0.0001$).

Figure 1 shows the performance at Baseline and Post-Shunt. The most marked improvements after shunt surgery were seen in learning, retention, and delayed recall of the RAVLT word list, and on the Trail Making Test, Part A. Except for recognition memory, there was significant improvement on all tests on which performance was impaired at Baseline ($>$ 1.5 s.d. below normal). On many of the remaining tests, including the Boston Naming Test, recall of the Rey Complex Figure, recall of the RAVLT interference list, and COWA word fluency, the mean performance was within the normal range at Baseline. This result is consistent with previous findings that the main cognitive skills that are affected in NPH are memory retrieval and executive functions. Although the COWA (word fluency) is often thought to be a test of executive function because it requires divergent thinking while maintaining task directions, switching tasks with each new letter, it does not appear to be as sensitive to the executive dysfunction observed in NPH as the Trail Making Test Part B. The mean performance on the Rey Complex Figure Delayed Recall was within normal limits at Baseline (in contrast to verbal recall); this finding likely reflects the very large standard deviation for normal elderly subjects on this test. Because of the wide variability in normal performance on this test, it is not an ideal test for identifying deficits associated with NPH or improvement after surgery. Recognition memory, thought to assess cortical function, was impaired at Baseline and did not improve after surgery. These results indicate that some patients may have had a cortical dementia in addition to the subcortical deficits of NPH.

Small average gains across tests of the type in Fig. 1 can reflect either small gains by the majority of patients or large gains by a minority of patients. In this study, since many patients performed within the normal range at Baseline, the changes reflect significant individual gains by the smaller subset of patients who were below the normal range at Baseline. Figure 2 shows the percentage of patients who performed within the normal, borderline impaired and definitely impaired ranges on each test at each condition. For example, on the WMS Logical Memory test (recalling stories) 36% were in the normal range at Baseline and 54% were in the normal range Post-Shunt. On the WMS Delayed Recall, the percentage in the normal range improved from 33% to 50%. Similarly, on learning (RAVLT total for five trials of list learning) the percentage of patients who performed within the normal range improved from 33%

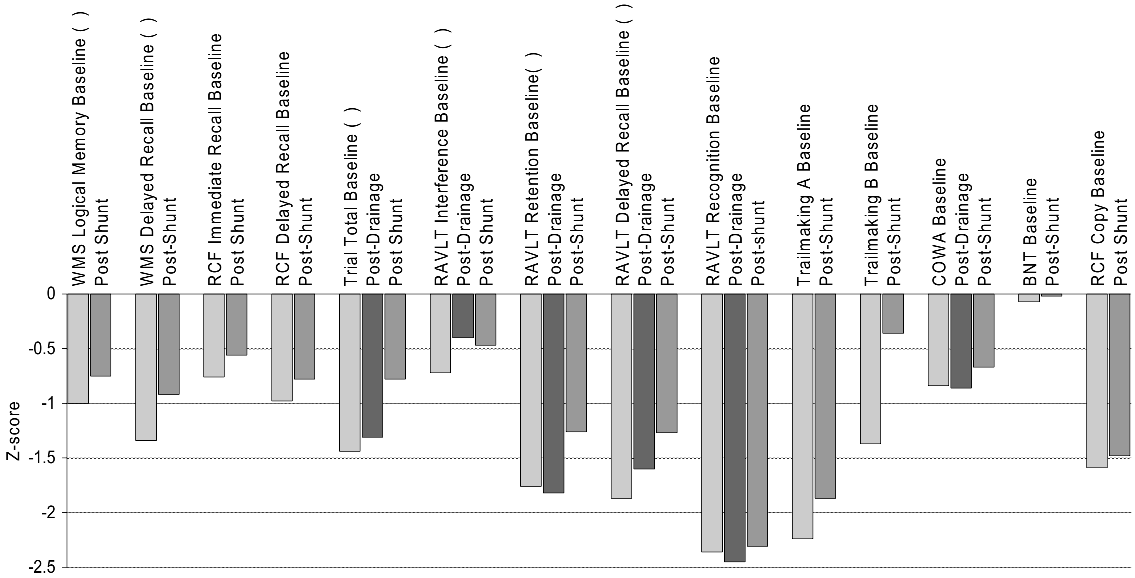

Fig. 1. Z scores for each test at each administration.

to 55%. Finally, although only 15% of patients performed within the normal range on the RAVLT Delayed Recall test at Baseline, 32% did so Post-Shunt. Likewise, on this test of delayed recall, the percentage who had definite deficits improved from 53% to 30% (Fig. 3). The majority (74%) of patients showed significant improvement by at least one standard deviation on at least one of the memory tests. There was very little change in the distribution scores on tests of executive function (COWA, Trail Making Test) from Baseline to Post-Shunt. Note, however, that approximately 50% of our patients performed within normal limits on these tests even at Baseline.

We next address the question of whether the magnitude of response to controlled lumbar drainage predicted the magnitude of response to surgery. On three subtests of the RAVLT, the degree of improvement in accuracy scores from Baseline to Post-Drainage significantly correlated with the degree of improvement in scores from Baseline to Post-Shunt: (1) learning ($r^2 = 0.58$; $p < 0.001$); (2) retention ($r^2 = 0.32$; $p = 0.04$); and (3) delayed recall ($r^2 = 0.36$; $p = 0.02$). There was also a significant association between improvement of at least 5 points on the RAVLT Learning test from Baseline to Post-Drainage and significant improvement on more than half of the memory tests from Baseline to Post-Shunt (chi squared $= 10.8$; $p = 0.0005$). The positive predictive value of 5-point improvement on this test was 50%; the negative predictive value was 96%. That is, patients who failed to show improvement of RAVLT learning scores from Baseline to Post-Drainage were very unlikely to improve significantly on the majority of memory tests three to six months after shunt insertion.

In regard to patient and family perception of cognitive impairment, information from the baseline questionnaire was available for 42 patients, of whom 37 (88%) perceived that they had "thinking and memory" problems. In regard to other INPH symptoms, 38/42 (90%) reported gait impairment and 30/42 (71%) reported urinary problems. As for changes in cognition after shunt surgery, information from post-shunt questionnaires (along with post-shunt cognitive testing) was available for 38 patients, of whom 14 (37%) perceived improvement in their cognitive function.

As for the relationship between patient and family perception of cognitive symptoms and cognitive testing results, none of the cognitive tests at baseline showed a consistent relationship with the patient's own perception of cognitive impairment. The largest areas under the curve (AUC) were 0.78 for the WMS delayed recall test and and 0.77 for the trailmaking A test. Similarly, there were no strong relationships between the patient's perception of cognitive improvement after shunt surgery and improvement in cognitive scores. The largest AUCs were 0.81 for the RCF delayed recall and and 0.69 for the RCF immediate recall test. All other AUCs for baseline and follow-up data were 0.70 or less.

Figure 4 shows that regardless of their actual performance on any of the cognitive tests administered, a

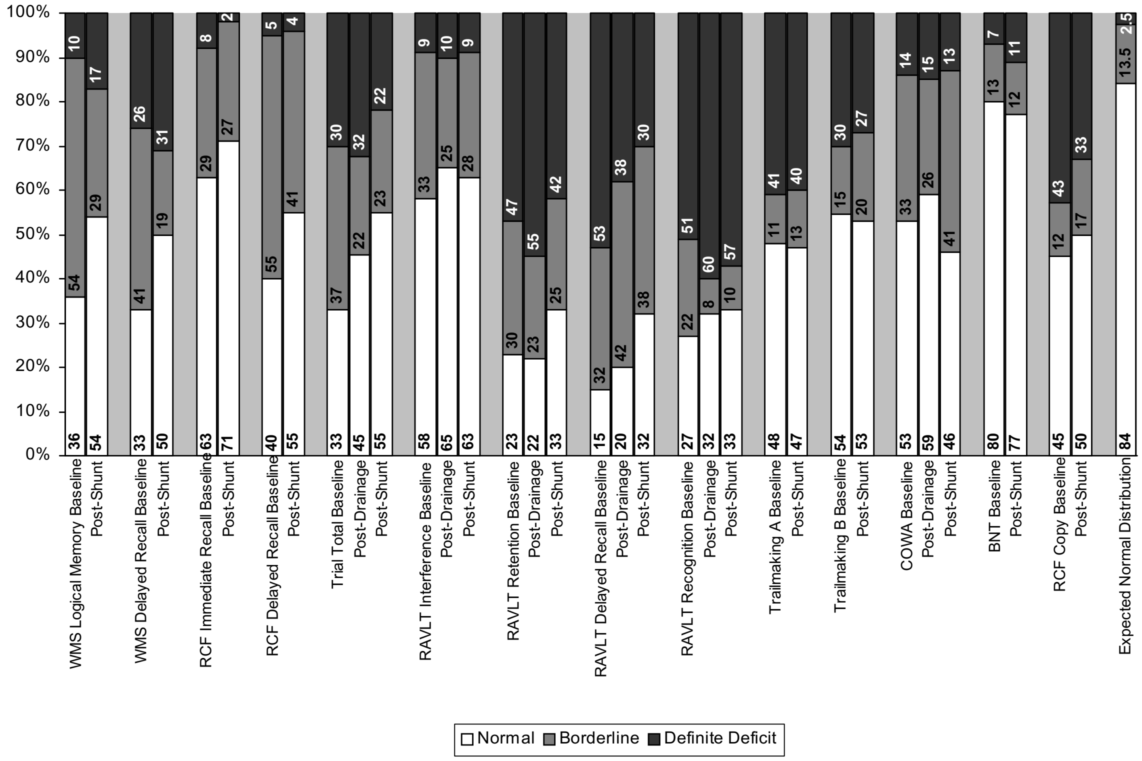

Fig. 2. Percentage of patients with each level of performance on each test at each administration.

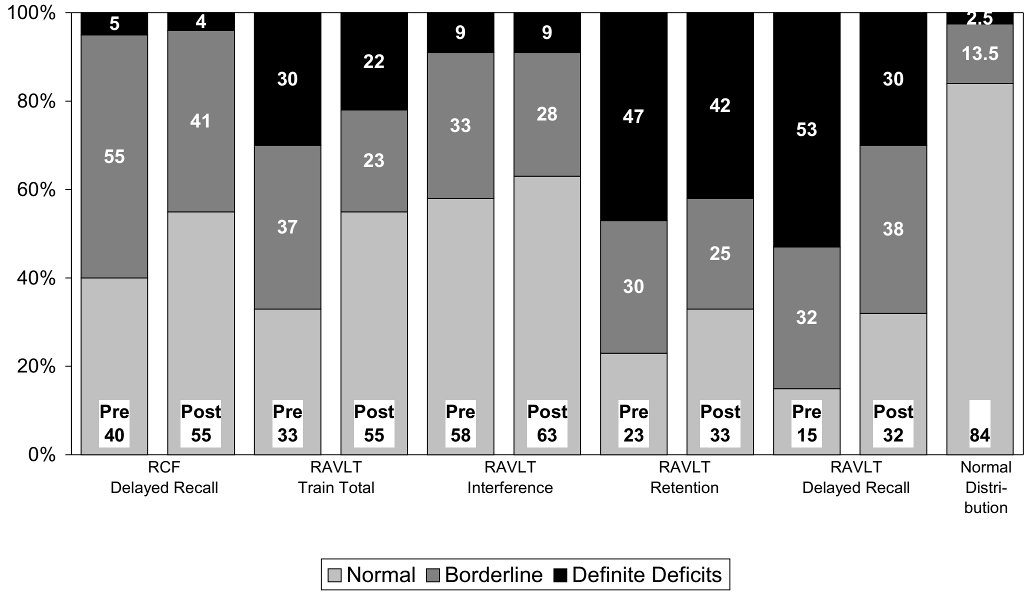

Fig. 3. Improvements in memory tests from baseline to post-shunt.

significant majority of patients felt they had cognitive impairment at baseline. Figure 5 shows the same lack of correlation between the patient's perception of cognitive improvement and objective change in any particular cognitive test after shunt surgery. As expected from the ROC analyses, the RCF immediate and RCF

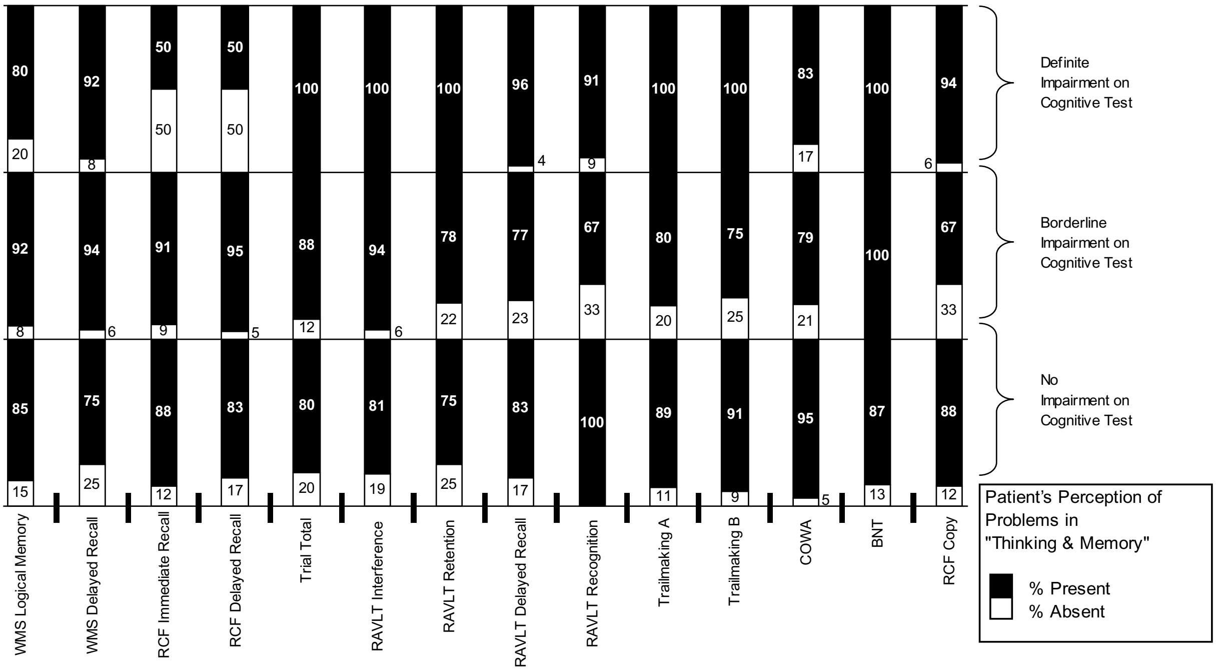

Definite Impairment on Cognitive Test
Borderline Impairment on Cognitive Test
No Impairment on Cognitive Test
Patient's Perception of Problems in "Thinking & Memory"
% Present
% Absent
WMS Logical Memory
WMS Delayed Recall
RCF Immediate Recall
RCF Delayed Recall
Trial Total
RAVLT Interference
RAVLT Retention
RAVLT Delayed Recall
RAVLT Recognition
Trailmaking A
Trailmaking B
COWA
BNT
RCF Copy

Fig. 4.

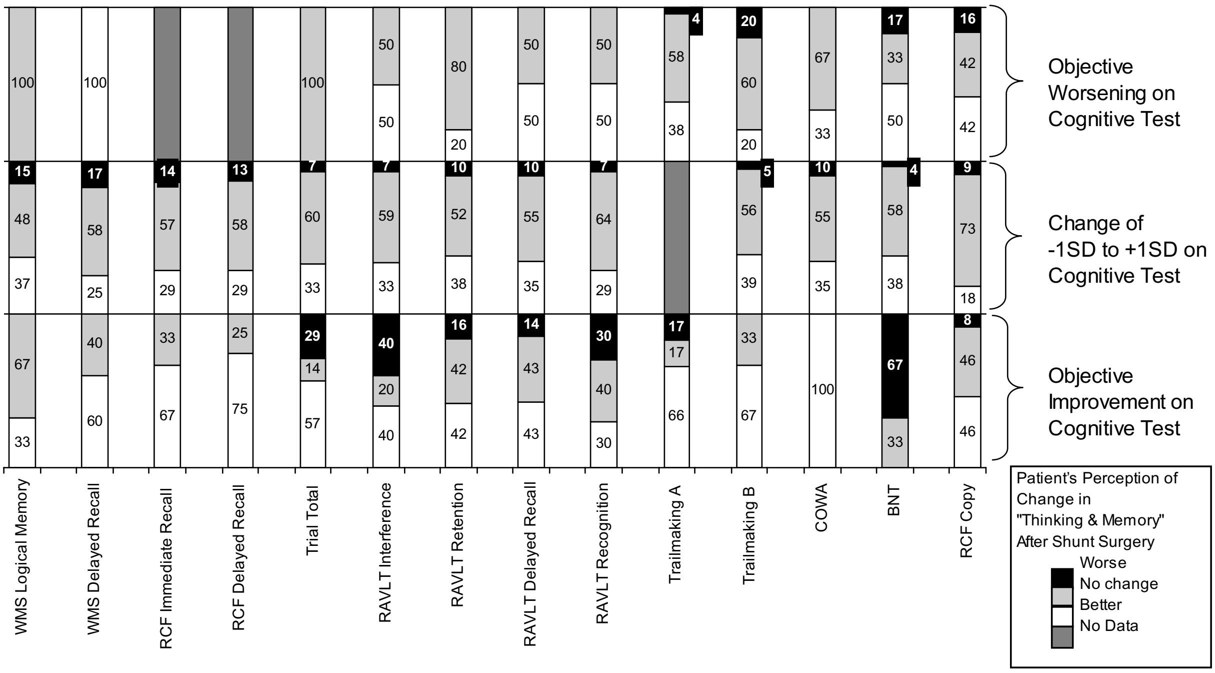

Objective Worsening on Cognitive Test
Change of -1SD to +1SD on Cognitive Test
Objective Improvement on Cognitive Test
Patient's Perception of Change in "Thinking & Memory" After Shunt Surgery
Worse
No change
Better
No Data
WMS Logical Memory
WMS Delayed Recall
RCF Immediate Recall
RCF Delayed Recall
Trial Total
RAVLT Interference
RAVLT Retention
RAVLT Delayed Recall
RAVLT Recognition
Trailmaking A
Trailmaking B
COWA
BNT
RCF Copy

Fig. 5.

delayed tests seemed to correspond most closely to the patient's subjective perception.

## 4. Discussion

One of the major challenges in the diagnosis and treatment of NPH is to identify which, if any, of the symptoms are likely to improve with shunt surgery, and the extent of recovery that might be expected. While there is good evidence that improvement of gait can be predicted based on the results of a trial of controlled CSF drainage [17,18], it has been more difficult to predict cognitive improvement. With this study, we were able to show that the magnitude of improvement following a trial of controlled CSF drainage significantly predicts the magnitude of improvement on the same tests 3–6 months after shunt insertion. In fact, most patients showed more improvement after shunt surgery than after the CSF drainage trial. While not all patients improved by 3–6 months after shunt surgery, it is probable that many of them would make gains later, especially considering that we tested patients sooner after shunt surgery in this study compared to our earlier studies that showed significant improvement at 6–12 months after shunt surgery [4,6,31].

As in previous studies, the most substantial improvements after shunt surgery were in learning new information and delayed recall, two of the most commonly reported deficits in NPH. Unlike other studies, we did not find a marked improvement in tests of executive functions, probably because of a ceiling effect, because about half our patients scored in the normal range at Baseline. More sensitive tests of executive function may be needed to demonstrate improvement on speed of processing in tests that require planning, sequencing, set shifting, and other executive functions.

Our previous studies were limited to patients who were referred for cognitive testing *because* of their cognitive symptoms. In this study, however, we attempted to include all patients who were referred for evaluation and treatment of NPH, regardless of the extent of cognitive impairment. Therefore, many had only impairment of gait and bladder control, and indeed had baseline cognitive testing that was within normal limits. Nevertheless, we were able to demonstrate significant improvements in the patients who were impaired at baseline, particularly in memory functions.

Taking our current study and prior work into account, we have found that significant predictors of cognitive improvement after shunt surgery for NPH include: (1) improvement on cognitive tests after a trial of controlled CSF drainage; (2) younger age [4]; (3) female sex [4]; (4) baseline WMS immediate recall score $<$ 1 s.d. below normal, especially if there are concurrent deficits in visuospatial or executive function [31], and (5) improvement in the RAVLT learning test by at least 5 points. However, some patients who would be expected not to improve on the basis of these factors nevertheless show significant improvement in scores on cognitive tests and report substantial gains in every day functioning.

The magnitude and speed of cognitive improvement after shunt surgery for NPH are not modest; they are substantial. The bulk of evidence from prospective, non-randomized studies indicates that at least 25% of patients improve substantially on most memory tests (e.g. at least 1 standard deviation in scores, or by 25% of raw scores) within 3-6 months of shunt surgery [4,6, 31] In contrast, none of the pharmacological interventions for Alzheimer disease have shown such significant improvement. A recent metanalysis of randomized placebo-controlled trials of cholinesterase inhibitors in Alzheimer's disease concluded that the proportion of people who responded to therapy was only 10 % (95% CI – 4 to 17%) higher in the treatment group as compared to the placebo group [14] Considering the amount of basic and clinical research into cognitive impairment and recovery in NPH is minuscule when compared to that of Alzheimer disease, we would argue that elucidation of the clinical patterns of cognitive impairment and recovery, and neural and glial mechanisms of injury and recovery in hydrocephalus, has the potential to benefit not only patients with NPH, but also patients with other causes of dementia if the injury and recovery mechanisms can be identified and generalized.

The issue of patient perception of cognitive impairment deserves further exploration. At baseline, 90% of patients perceived that they had problems with cognition, which contrasts sharply with studies of Alzheimer dementia (AD), in which many patients report no cognitive impairment despite clear objective evidence to the contrary [12,13,29] Whether patient perception of cognitive impairment can be used to distinguish INPH from AD remains to be determined.

Despite their ability to perceive baseline cognitive impairment, patients with INPH seem to be less adept at perceiving improvement in cognitive function after shunt surgery. Only 37% perceived that their "thinking and memory" had improved after shunt surgery. Their perception of improvement seemed to correspond best with tests for memory; however, no particular test could completely predict patient response at any cut-off. We postulate that the main reason for this is that cognitive recovery in NPH occurs in different cognitive domains in different patients, so that no particular cognitive domain improves in all patients who after shunt surgery feel that their cognitive symptoms have been "successfully treated". Their limited ability to perceive cognitive improvement may be an additional reason to administer a cognitive battery after shunt surgery, so as to document change and offer reassurance when improvement has occurred, or to identify that impairment persists and begin seeking other etiologies of dementia.

The main weakness of this and virtually all other studies of NPH is that we did not have a control group of patients with NPH who were followed without shunt surgery. This raises the question of whether there is a significant placebo response to treatment in NPH, as has been identified in Parkinson's disease for medical therapy with the DATATOP study [7] and surgical therapy with a sham surgery control, as with transplantation of human embryonic dopamine neurons into the brain, in which patients who thought they were receiving the cell transplant (regardless of their actual assignment to transplant or sham) reported better quality of life scores [19]. A placebo response in the Alzheimer's Disease Assessment Scale has also been noted and seems to be greatest for those tasks most susceptible to practice effect, such as word recall and naming objects [5] In our study, we used alternate forms of the Wechsler Memory logical memory test, COWA, and RAVLT to minimize practice effect. The fact that the magnitude of cognitive improvement is dramatically better with shunt surgery in NPH than with pharmacologic therapy of Alzheimer disease might argue against a placebo effect; however, it has been suggested that the magnitude of the placebo effect should be greater for surgical as opposed to pharmacologic interventions [19].

Future well-designed studies are required to confirm that clinical improvement in NPH is due to the surgical intervention. Such a study would require careful design, including double-blinding, and ethical safeguards to protect patients from harm either because they might receive shunt surgery when they shouldn't, or might not receive shunt surgery when they should. Furthermore, because shunt surgery has been an established treatment for NPH for over 40 years, it is conceivable that it would be difficult to enroll patients in a trial with sham surgery, as they could easily seek shunt surgery elsewhere, unlike Phase II clinical trials or the cell transplant study for Parkinsonism, in which the treatment is truly experimental and cannot be obtained elsewhere [19]. Lastly, a study with a control group, or a cross-over design study would need to incorporate a sham shunt designed in such a way that it could be converted to a functional shunt implant as non-invasively as possible, so as to minimize the operative and perioperative risk to research subjects.

## References

[1] R.D. Adams, C.M. Fisher, S. Hakim, R.G. Ojemann and W.H. Sweet, Symptomatic Occult Hydrocephalus with "Normal" Cerebrospinal-fluid pressure: A treatable syndrome, *N Engl J Med* **273** (1965), 117–126.

[2] G. Aygok, A. Marmarou and H.F. Young, Three-year outcome of shunted idiopathic NPH patients, *Acta Neurochir Suppl* **95** (2005), 241–245.

[3] M. Bergsneider, P.M. Black, P. Klinge, A. Marmarou and N. Relkin, Surgical management of idiopathic normal-pressure hydrocephalus, *Neurosurgery* **57** (2005), S29–S39.

[4] S. Chang, S. Agarwal, M.A. Williams, D. Rigamonti and A.E. Hillis, Demographic factors influence cognitive recovery after shunt for normal-pressure hydrocephalus, *Neurologist* **12** (2006), 39–42.

[5] P.M. Doraiswamy, L. Kaiser, F. Bieber and R.L. Garman, The Alzheimer's Disease Assessment Scale: evaluation of psychometric properties and patterns of cognitive decline in multicenter clinical trials of mild to moderate Alzheimer's disease, *Alzheimer Dis Assoc Disord* **15** (2001), 174–183.

[6] A. Duinkerke, M.A. Williams, D. Rigamonti and A.E. Hillis, Cognitive recovery in idiopathic normal pressure hydrocephalus after shunt, *Cogn Behav Neurol* **17** (2004), 179–184.

[7] C.G. Goetz, S. Leurgans and R. Raman, Placebo-associated improvements in motor function: comparison of subjective and objective sections of the UPDRS in early Parkinson's disease, *Mov Disord* **17** (2002), 283–288.

[8] J.C. Golden, *Stroop Colour and Word Test*, Sterling Co, Chicago, 1978.

[9] H. Goodglass, E. Kaplan and S. Weintraub, *The Revised Boston Naming Test*, Lea & Febiger, Philadelphia, 1983.

[10] L. Gustafson and B. Hagberg, Recovery in hydrocephalic dementia after shunt operation, *J Neurol Neurosurg Psychiatry* **41** (1978), 940–947.

[11] S. Hakim and R.D. Adams, The special clinical problem of symptomatic hydrocephalus with normal cerebrospinal fluid pressure – Observations on cerebrospinal fluid hydrodynamics, *J Neurol Sci* **2** (1965), 307–327.

[12] D.G. Harwood, D.L. Sultzer and M.V. Wheatley, Impaired insight in Alzheimer disease: association with cognitive deficits, psychiatric symptoms, and behavioral disturbances, *Neuropsychiatry Neuropsychol Behav Neurol* **13** (2000), 83–88.

[13] Y. Kashiwa, Y. Kitabayashi, J. Narumoto, K. Nakamura, H. Ueda and K. Fukui, Anosognosia in Alzheimer's disease: association with patient characteristics, psychiatric symptoms and cognitive deficits, *Psychiatry Clin Neurosci* **59** (2005), 697–704.

[14] K.L. Lanctot, N. Herrmann, K.K. Yau, L.R. Khan, B.A. Liu, M.M. LouLou and T.R. Einarson, Efficacy and safety of cholinesterase inhibitors in Alzheimer's disease: a meta-analysis, *Can Med Assoc J* **169** (2003), 557–564.

[15] A. Larsson, C. Wikkelso, M. Bilting and H. Stephensen, Clinical parameters in 74 consecutive patients shunt operated for normal pressure hydrocephalus, *Acta Neurol Scand* **84** (1991), 475–482.
[16] J. Malm, B. Kristensen, T. Karlsson, M. Fagerlund, J. Elfverson and J. Ekstedt, The predictive value of cerebrospinal fluid dynamic tests in patients with the idiopathic adult hydrocephalus syndrome, *Arch Neurol* **52** (1995), 783–789.
[17] A. Marmarou, M. Bergsneider, P. Klinge, N. Relkin and P.M. Black, The value of supplemental prognostic tests for the preoperative assessment of idiopathic normal-pressure hydrocephalus, *Neurosurgery* **57** (2005), S17–S28.
[18] M.J. McGirt, G. Woodworth, A.L. Coon, G. Thomas, M.A. Williams and D. Rigamonti, Diagnosis, treatment, and analysis of long-term outcomes in idiopathic normal-pressure hydrocephalus, *Neurosurgery* **57** (2005), 699–705.
[19] C. McRae, E. Cherin, T.G. Yamazaki, G. Diem, A.H. Vo, D. Russell, J.H. Ellgring, S. Fahn, P. Greene, S. Dillon, H. Winfield, K.B. Bjugstad and C.R. Freed, Effects of perceived treatment on quality of life and medical outcomes in a double-blind placebo surgery trial, *Arch Gen Psychiatry* **61** (2004), 412–420.
[20] A. Ogino, H. Kazui, N. Miyoshi, M. Hashimoto, S. Ohkawa, H. Tokunaga, Y. Ikejiri and M. Takeda, Cognitive impairment in patients with idiopathic normal pressure hydrocephalus, *Dement Geriatr Cogn Disord* **21** (2006), 113–119.
[21] P.A. Osterrieth, Le test de copie d'une figure complex: contribution a l'étude de la perception et de la mémoire, *Arch Psychol* **30** (1944), 286–356.
[22] J.E. Partington and R.G. Leiter, Partington's pathway test, *Psychological Service Center Bulletin* **1** (1949), 9–20.
[23] M.A. Poca, M. Mataro, M. Matarin, F. Arikan, C. Junque and J. Sahuquillo, Good outcome in patients with normal-pressure hydrocephalus and factors indicating poor prognosis, *J Neurosurg* **103** (2005), 455–463.
[24] C. Raftopoulos, J. Deleval, C. Chaskis, A. Leonard, F. Cantraine, F. Desmyttere, S. Clarysse and J. Brotchi, Cognitive recovery in idiopathic normal pressure hydrocephalus: a prospective study, *Neurosurgery* **35** (1994), 397–404.
[25] N. Relkin, A. Marmarou, P. Klinge, M. Bergsneider, and P.M. Black, Diagnosing idiopathic normal-pressure hydrocephalus, *Neurosurgery* **57** (2005), S4–16.
[26] T. Sand, G. Bovim, R. Grimse, G. Myhr, G. Helde and J. Cappelen, Idiopathic normal pressure hydrocephalus: the CSF tap-test may predict the clinical response to shunting, *Acta Neurol Scand* **89** (1994), 311–316.
[27] S. Savolainen, H. Hurskainen, L. Paljarvi, I. Alafuzoff and M. Vapalahti, Five-year outcome of normal pressure hydrocephalus with or without a shunt: predictive value of the clinical signs, neuropsychological evaluation and infusion test, *Acta Neurochir* **144** (2002), 515–523.
[28] M. Schmidt, *Rey Auditory Verbal Learning Test* (*RAVLT*) – *A Handbook*, Western Psychological Services, Los Angeles, 1996.
[29] S. Sevush, Relationship between denial of memory deficit and dementia severity in Alzheimer disease, *Neuropsychiatry Neuropsychol Behav Neurol* **12** (1999), 88–94.
[30] O. Spreen and A.L. Benton, *Neurosensory Center Comprehensive Examination for Aphasia* (*NCCEA*), University of Victoria Neuropsychology Laboratory, Victoria, 1969.
[31] G. Thomas, M.J. McGirt, G. Woodworth, J. Heidler, D. Rigamonti, A.E. Hillis and M.A. Williams, Baseline neuropsychological profile and cognitive response to cerebrospinal fluid shunting for idiopathic normal pressure hydrocephalus, *Dement Geriatr Cogn Disord* **20** (2005), 163–168.
[32] C.N. Tromp, M.J. Staal and L.E. Kalma, Effects of ventricular shunt treatment of normal pressure hydrocephalus on psychological functions, *Z Kinderchir* **44**(Suppl 1) (1989), 41–43.
[33] J.A. Vanneste, Diagnosis and management of normal-pressure hydrocephalus, *J Neurol* **247** (2000), 5–14.
[34] D. Weschler, *The Weschler Memory Scale – Revised*, Psychological Corporation, New York, 1987.
